# β2-microglobulin induces epithelial-mesenchymal transition in human renal proximal tubule epithelial cells in vitro

**DOI:** 10.1186/s12882-015-0057-x

**Published:** 2015-04-23

**Authors:** Aiqing Zhang, Bin Wang, Min Yang, Huimin Shi, Weihua Gan

**Affiliations:** Department of Pediatric Nephrology, the Second Affiliated Hospital of Nanjing Medical University, Nanjing, 210011 P. R. China; Division of Nephrology, Huashan Hospital and Institute of Nephrology, Fudan University, Shanghai, 200040 P.R. China; Department of Nephrology, the Third Affiliated Hospital of Soochow University, Changzhou, 210003 China

**Keywords:** β2-microglobulin, Epithelial-mesenchymal transition, Hemochromatosis

## Abstract

**Background:**

The objective of this study was to investigate the influence of β2-microglobulin (β2-M) on the epithelial-mesenchymal transition (EMT) in renal tubular epithelial cells.

**Methods:**

A human kidney proximal tubular cell line (HK-2) was used as the proximal tubular cell model. HK-2 cells were exposed to different concentrations of β2-M (5, 10, 25, and 50 μM) for up to 24, 48 and 72 h. The effects of β2-M on cell morphology were observed by phase contrast microscopy, and the possible associated mechanisms were assessed by immunofluorescence staining, western blot, RNA interference, immunoprecipitation, and induced coupled plasma mass spectroscopy.

**Results:**

β2-M induced marked morphological alterations in the HK-2 cells, accompanied by the increased expression of extracellular matrix components and α-smooth muscle actin (α-SMA), vimentin and fibronectin and the reduced expression of E-cadherin. Our results also revealed that β2-M could induce the EMT in the HK-2 cells without significant affecting cell viability. Excess β2-M in the HK-2 cells led to a decrease in iron and an increase in hypoxia inducible factor-1α (HIF-1α), which induced EMT in the HK-2 cells. Additionally, disrupting the function of the β2-M/hemochromatosis (HFE) complex by HFE knockdown was sufficient to reverse β2-M-mediated EMT in the HK-2 cells.

**Conclusion:**

These findings demonstrate that the activity of β2-M is mediated by the β2-M/HFE complex, which regulates intracellular iron homeostasis and HIF-1α and ultimately induces EMT in HK2 cells.

## Background

Epithelial-mesenchymal transition (EMT) is a process by which fully differentiated epithelial cells undergo transition to a fibroblast phenotype. Emerging evidence has suggested that EMT is an important pathway leading to the generation of matrix-producing fibroblasts and myofibroblasts during the development of kidney fibrosis [1-5].

Protein overload models as well as animal models of proteinuria induced by renal injury have suggested that excessive protein reabsorption induces EMT in tubular cells [6,7]. Qiong Wen et al. [8] have proven that urinary proteins from patients with nephrotic syndrome alter signaling proteins regulating EMT. *In vitro* experiments have found that a high concentration of albumin up-regulates profibrogenic gene (transforming growth factor-β1, TGF-β1) expression in proximal tubular cells [9].

β2-microglobulin (β2-M) is an 11-kDa nonglycosylated protein with no transmembrane domain that usually associates with cells by interacting with the extracellular regions of heavy chains. It is typically filtered through the renal glomeruli, and 99% of it is absorbed and catalyzed by the renal tubular cells, while the non-absorbed portion is excreted in the urine. β2-M is also a major protein component of proteinuria. The appearance of β2-M in the urine depends on its plasma level, which must exceed its renal reabsorptive threshold of 5 mg/l, and/or proximal tubular damage [10]. Many studies have demonstrated that the serum or urine β2-M concentration is increased in a variety of diseases, including inflammatory or infectious diseases [11,12], prostate cancer, lung cancer, and particularly in lymphocytic malignancies, such as non-Hodgkin’s lymphoma and multiple myeloma [13-17]. However, the effect of excessive β2-M on proximal tubular cells is relatively unknown.

Therefore, the main aim of this study was to test the hypothesis that urinary β2-M can induce EMT human renal proximal tubule epithelial cells and to elucidate the molecular mechanism associated with this process.

## Methods

### Cell cultures and treatments

HK-2 human renal proximal tubular epithelial cells (PTECs) were purchased from the American Type Culture Collection (Rockville, MD) and were maintained as described previously [8]. A β2-M stock solution (1 mM) was prepared by dissolving 118 mg of lyophilized powder into 10 ml of serum-free base media. Human serum albumin (HSA), which is the most abundant protein in nephrotic urine, was used as a protein control. We used TGF-β1, which is a classic profibrogenic factor, as a positive control. The HK-2 cells were exposed to different concentrations of β2-M, HAS, or TGF-β1 for various periods of time as scheduled. For neutralizing antibody assessments, cells were pre-treated for 1 h prior to the addition of β2-M and subsequently co-treated with a monoclonal anti-TGF-β1 antibody (TGF-β1 mAb; R&D Systems, Minneapolis, MN) at a concentration of 30 μg/ml.

### Cell viability and preparation of cell lysates

After exposure to the β2-M (5, 10, 25, and 50 μM) or control medium for 24–72 h, cell viability was measured by a Cell Titer 96 Aqueous One solution Cell Proliferation Assay (Promega, Madison, MI). To evaluate the transition of PTECs to fibroblasts, we used HK-2 cells cultured in supplement-free fresh media in the presence or absence of various treatments. After the media were removed, cellular proteins were extracted by lysing the cells with Mammalian Cell Lysis Reagents (Sigma). The protein concentrations in the cellular lysates were determined by the Bradford method using DC protein assay reagents (Bio-Rad, Hercules, CA).

### Western blot analysis

Western blot analysis was performed essentially according to an established procedure [18]. The primary antibodies used were as follows: anti-β-actin (Sigma, St. Louis, MO), anti-α-SMA (Sigma, St. Louis, MO), anti-E-cadherin (BD Transduction Laboratories, Lexington, KY), anti-fibronectin (Santa Cruz Biotechnology, Santa Cruz, CA), anti-HFE (Santa Cruz Biotechnology, Santa Cruz, CA), anti-vimentin (Boster, Wuhan, China), and anti-HIF-1α (Millipore, USA). Quantifications were performed by measuring band intensities using Image J analysis software.

### Transient transfection of cells with siRNA

For the knockdown experiments, HK2 cells were transiently transfected with siRNA specifically targeting HFE or a negative control siRNA using Lipofectamine PLUS (Invitrogen, Carlsbad, CA) according to the manufacturer’s instructions. Cells were transfected with 20 nM HFE siRNA or control siRNA for 24 h before treatment. Cellular protein was extracted and subjected to western blot analysis for HFE.

### Immunoprecipitation

Immunoprecipitation was performed using an Immunoprecipitation Starter Pack (GE Healthcare).

### Iron measurements and Iron chelator treatments on HK-2 cells

Iron concentrations were determined using induced coupled plasma mass spectroscopy (ICP-MS). A total of 10^7^ cells were pelleted and digested using 3% nitric acid. Samples were diluted and analyzed with a Perkin Elmer ICP-MS. The data are expressed as picomoles of metal. HK-2 cells were treated with 200 mmol/L of desferal (DES) for 48 h. Then, the DES was removed and replaced with normal media. A day later, the cells were photographed, and cell lysates were prepared for immunoblot analysis.

### Statistical analysis

All data are expressed as the mean ± S.D. Multiple groups of values were compared using one-way analysis of variance (ANOVA) and Fisher’s LSD test. Data were analyzed using SPSS 13.0 for Windows. A P < 0.05 was considered significant.

## Results

### Effect of β2-M on PTEC cellular viability

**Figure 1:**
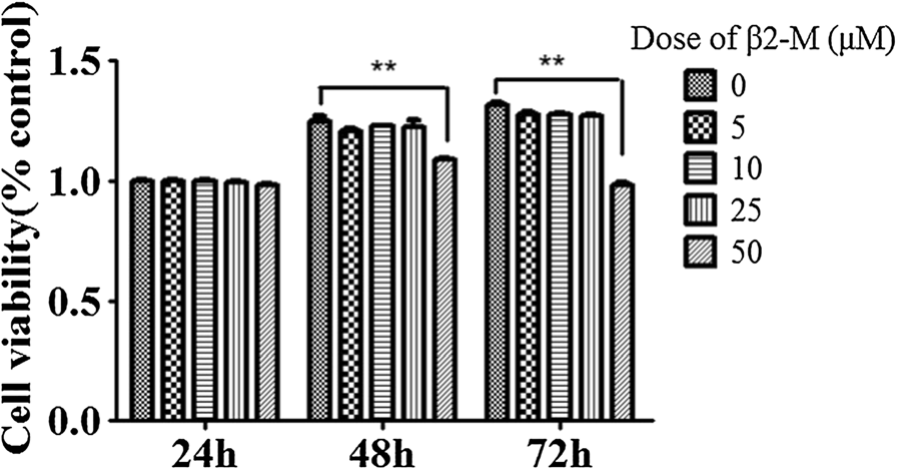
Effects of β2-M treatment on the viability of HK-2 cells. HK-2 cells were grown to 80% confluence, and then, different concentrations of β2-M (5, 10, 25, and 50 μM) were added to the media for an additional 24, 48, and 72 h. The mean and SE of the absorbance at 490 nm for six replicates normalized to a control was calculated for each group, considering the absorbance at 490 nm of the control as 1. **P < 0.05 compared with the control.

Considering the absorbance at 490 nm in the control group as 1, the exposure of HK-2 cells to different concentrations of β2-M (5, 10, and 25 μM) produced no significant effects on cell viability at 24, 48 or 72 h; however, a significant decrease in cell viability (>20%) was observed at the higher doses of ≥50 μM β2-M after 48 h (Figure 1).

### Effect of β2-M on PTEC morphology

**Figure 2:**
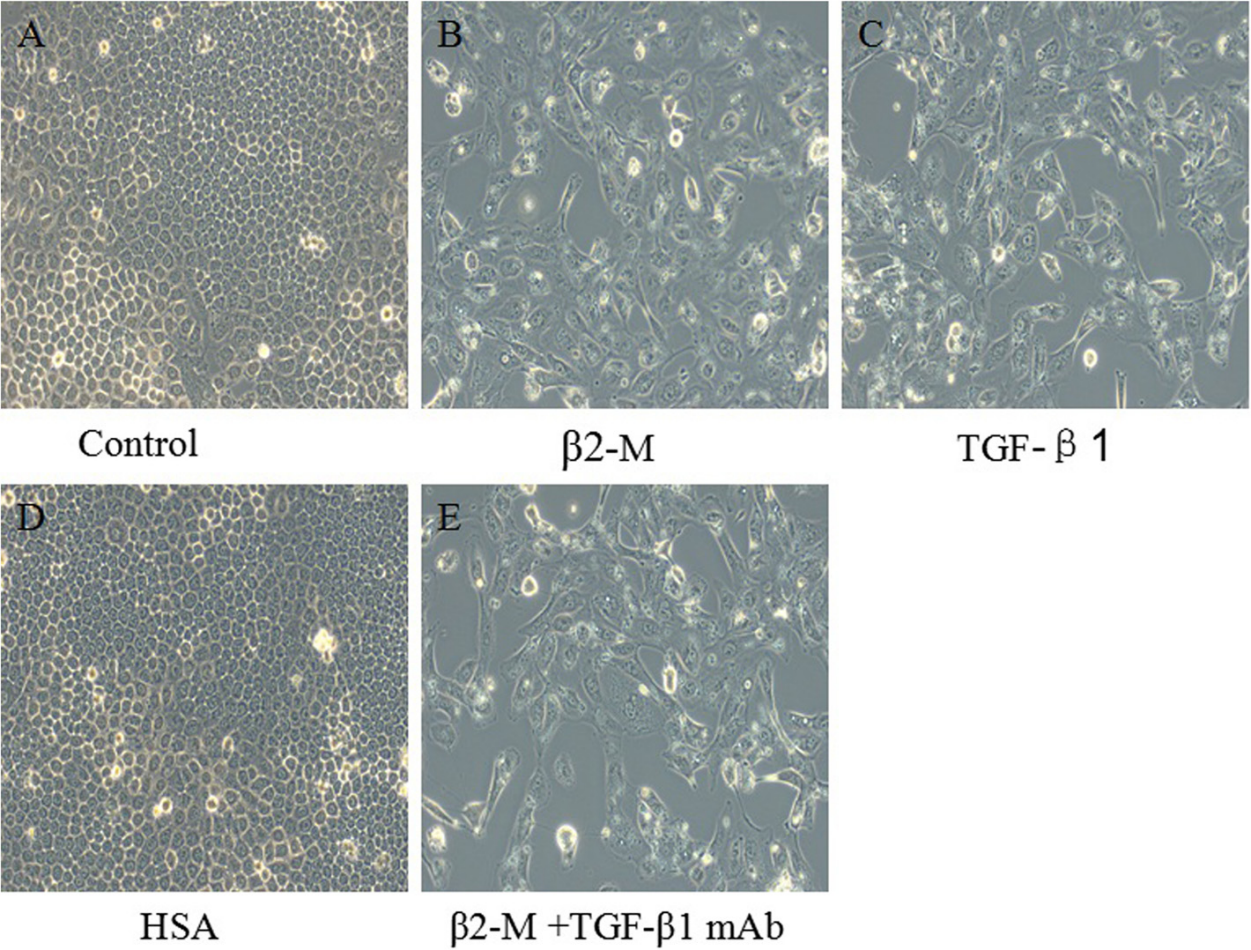
Effect of β2-M treatment on the morphology of HK-2 cells. HK-2 cells were grown in 6-well plates to 80% confluence, and then, control medium **(A)**, β_2_-M **(B)**, TGF-β_1_ **(C)**, HSA **(D)**, or β_2_-M + TGF-β_1_ mAb **(E)** was added for 72 h. The cells were photographed using a JVC KY-FSSBE digital camera coupled to a Nikon TMS microscope. For each group, 80 cells were measured.

The control cells showed a typical epithelial cuboidal shape with a cobblestone morphology (Figure 2A); however, they exhibited profound morphologic changes after exposure to β2-M (25 μM) for 72 h, becoming elongated in shape, disassociating from neighboring cells and losing their cobblestone monolayer morphology (Figure 2B). Because TGF-β1 is a well-characterized inducer of EMT in renal tubular epithelial cells, it was used as a positive control (Figure 2C). HSA, which was used as a protein control, had no significant effect on the morphological changes of the HK2 cells at 50 μM (Figure 2D). More interestingly, a neutralizing TGF-β1 antibody could not block the β2-M-induced morphological changes of the HK2 cells (Figure 2E), indicating that β2-M EMT occurred in a TGF-β1-independent manner.

### β2-M induced EMT in HK-2 cells

**Figure 3:**
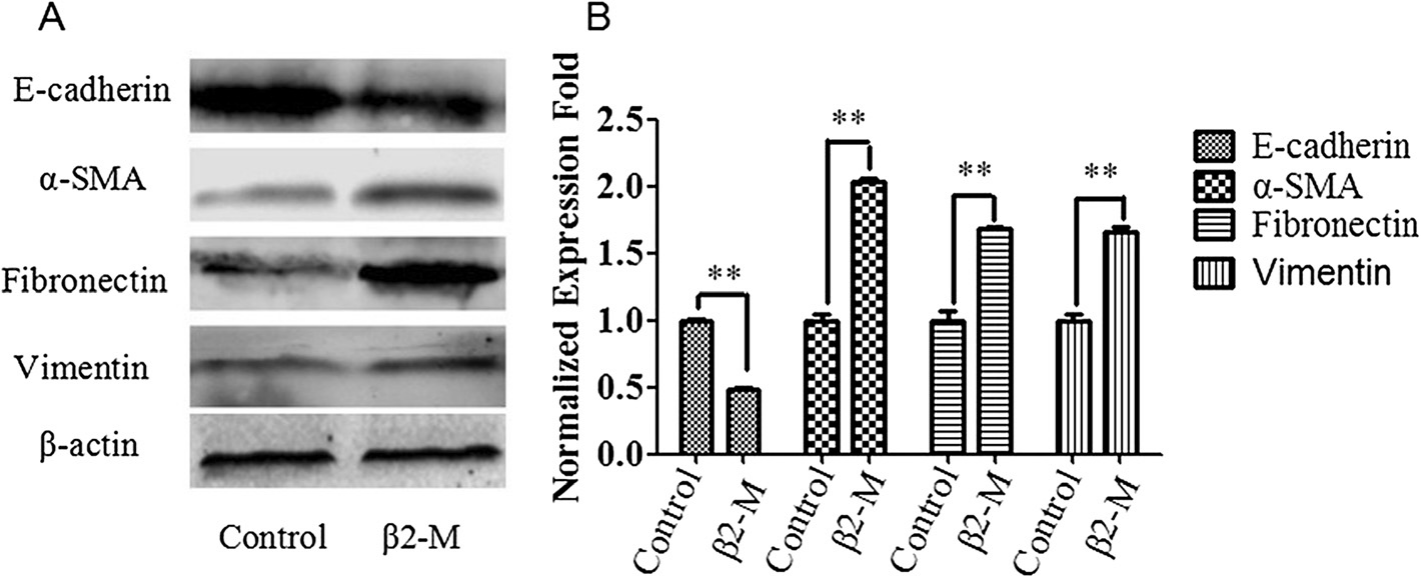
Effect of β2-M treatment on EMT in HK-2 cells. **(A)** HK-2 cells were stimulated with 25 μM β2-M for 72 h, Cell lysates were immunoblotted with antibodies against E-cadherin, α-SMA, fibronectin, vimentin, and β-actin. **(B)** Graphical representation of E-cadherin, α-SMA, vimentin, and fibronectin protein abundances as indicated. The relative E-cadherin, α-SMA, vimentin, and fibronectin levels were calculated after normalization to β-actin. The data are presented as the mean ± SE of three experiments. **P < 0.05 versus controls.

We further assessed the expression of E-cadherin, which is a marker for the epithelial phenotype, and α-SMA, vimentin, and fibronectin, which are markers for the myofibroblast phenotype. We observed that in HK-2 cells exposed to 25 μM of β2-M, the expression of E-cadherin decreased after 72 h of treatment, while that of α-SMA, vimentin, and fibronectin increased, as indicated by western blot analysis (Figure 3A and B), These results suggest that these cells lost their epithelial characteristics and acquired mesenchymal cell properties.

### β2-M interacted with hemochromatosis (HFE) protein and inhibition of HFE reversed EMT

**Figure 4:**
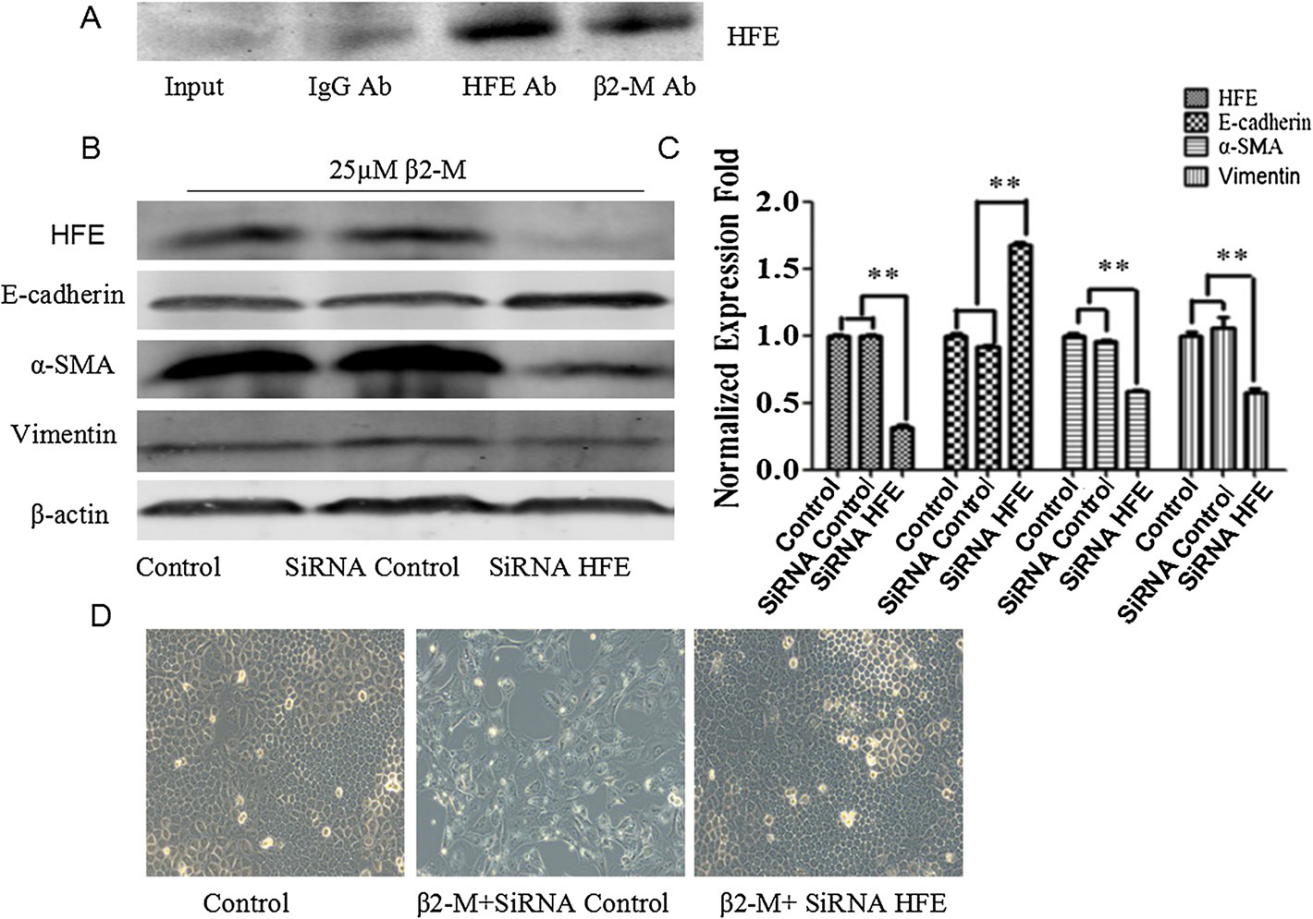
The β2-M-induced inhibition of HFE reversed the EMT process. **(A)** Immunoprecipitation using a monoclonal anti-β2-M Ab and an anti-HFE Ab. Western blot analysis of HFE. An IgG Ab was used as a control. **(B)** Western blot analysis of HFE and EMT markers (E-cadherin, vimentin, and α-SMA) in control and HFE knockdown HK-2 cells. **(C)** Graphical representation of relative HFE, E-cadherin, vimentin, and α-SMA protein abundances after normalization with β-actin as indicated. The data are presented as the mean ± SE of three experiments. **P < 0.05 versus controls. **(D)** HFE knockdown in HK-2 cells blocked β2-M activity and induced epithelial-like phenotypic changes. Photographs were taken using a Nikon TMS microscope at 400x magnification.

HFE has been previously shown to interact with β2-M. We therefore assessed the presence of β2-M and HFE complexes in HK-2 cells. The physical interaction between β2-M and HFE as a complex was demonstrated by coimmunoprecipitation (co-IP) followed by western blot analyses (Figure 4A). To determine the possible functional roles of the β2-M/HFE complex in β2-M-induced EMT in HK-2 cells, we transfected these cells with either siRNA-HFE or irrelevant siRNA-con. After 24 h, HFE expression was assessed by western blot. Our results indicated that the level of this protein was markedly lower in the HK-2 cells transfected with siRNA-HFE compared with the control cells and those transfected with siRNA-con (Figure 4B). Interestingly, a decrease in the HFE protein level blocked the increased expression of vimentin and α-SMA and decreased expression of E-cadherin induced by β2-M (Figure 4B and C). Additionally, we observed that the down-regulation of the HFE protein altered the morphology of the HK-2 cells to a cobblestone-like appearance (Figure 4D). Thus, disrupting the function of the β2-M/HFE complex by HFE knockdown is sufficient to reverse β2-M-mediated EMT in HK-2 cells.

### β2-M attenuated intracellular iron but increased HIF-1α expression in HK-2 cells

**Figure 5:**
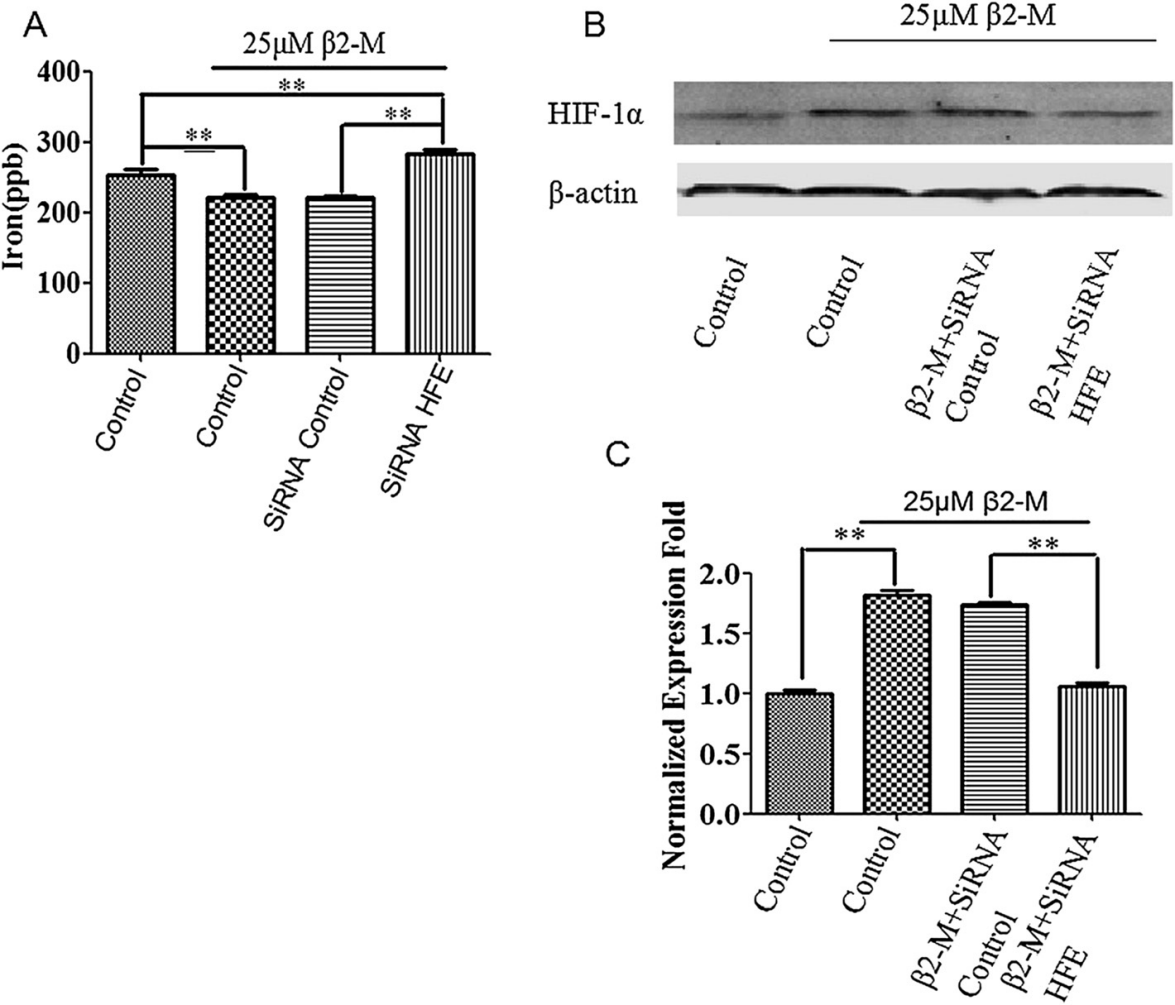
The β2-M/HFE complex modulated iron levels and induced HIF-1α expression. **(A)** Iron levels in HK-2 cells and HFE knockdown cells were measured by inductively coupled plasma mass spectroscopy. **(B)** Western blot analysis of HIF-1α in HK-2 cells and HFE knockdown cells. **(C)** Graphical representation of relative HIF-1α protein abundances after normalization with β-actin as indicated. The data are presented as the mean ± SE of three experiments. **P < 0.05 versus controls.

Because the β2-M protein is known to directly regulate iron levels in cells as demonstrated by the prevention of iron uptake by the β2-M/HFE complex and the iron overload observed in β2-M and HFE knockout mice (20), we assessed whether cellular iron levels were lower in β2-M-stimulated HK-2 cells compared to control cells using inductively coupled plasma mass spectroscopy (ICP-MS). Intracellular iron was significantly lower in the β2-M-stimulated HK-2 cells compared to the control cells (Figure 5A). Additionally, iron-responsive HIF-1α, which is a factor that has been widely proven to participate in the EMT process, increased after stimulation with β2-M for 72 h; however, HFE knockdown reversed the increase in HIF-1α induced by β2-M (Figure 5B and C).

### Iron modulated EMT in HK-2 cells

**Figure 6:**
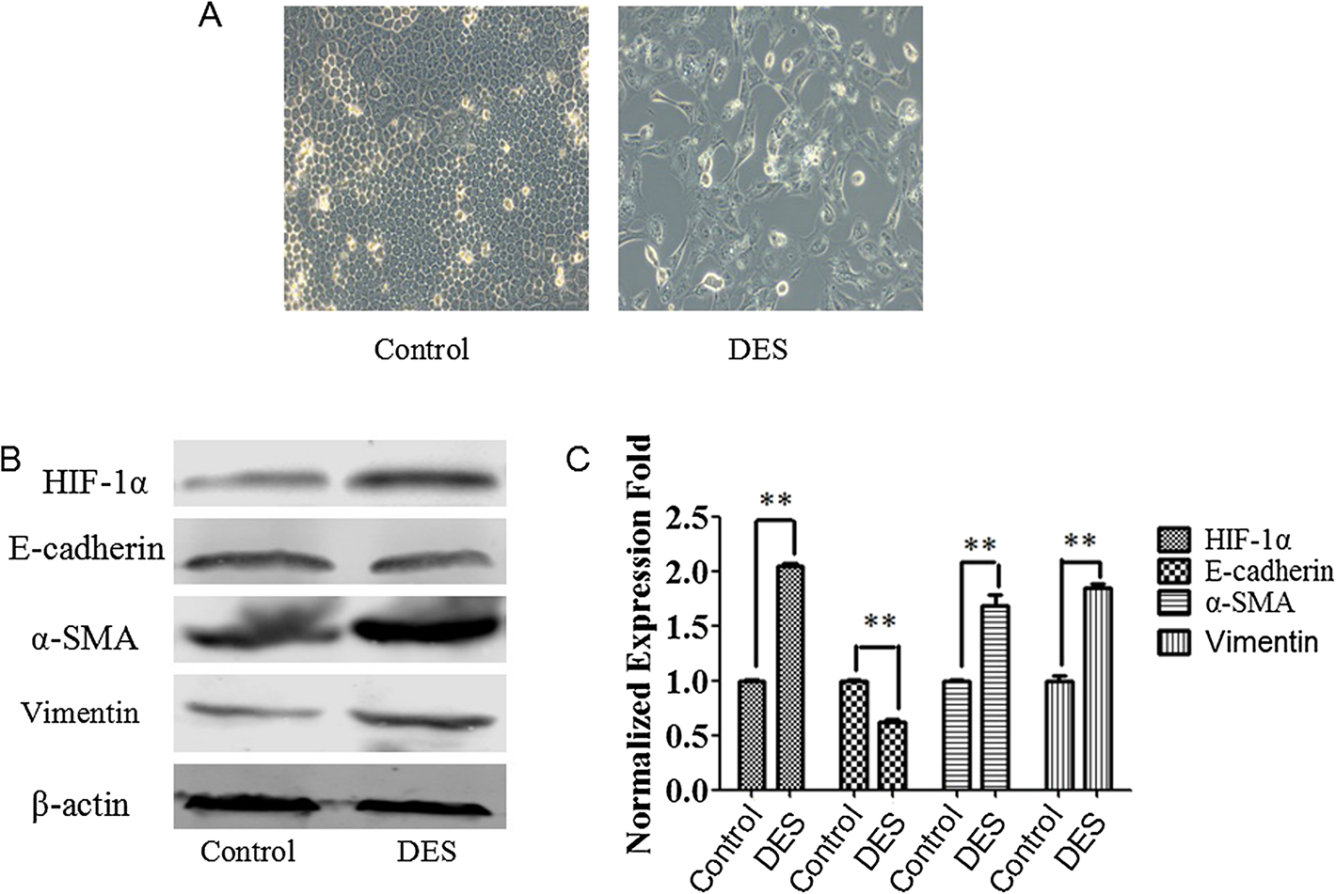
Effect of iron on EMT in HK-2 cells. **(A)** HK-2 cells were stimulated with DES for 48 h, and photographs were taken using a Nikon TMS microscope at 400x magnification. **(B)** HK-2 cells were cultured in the presence or absence of DES for 48 h. Cell lysates were immunoblotted with antibodies against HIF-1α, E-cadherin, vimentin, and α-SMA. **(C)** Graphical representation of relative HIF-1α, E-cadherin, vimentin, and α-SMA protein abundances after normalization with β-actin as indicated. The data are presented as the mean ± SE of three experiments. **P < 0.05 versus controls.

To further determine whether iron is involved in the regulation of EMT, we used an iron chelator (DES) to induce EMT-like changes. DES increased the expression of iron responsive HIF-1α and induced mesenchymal characteristics (Figure 6A through C). These results collectively demonstrate that β2-M overload in HK-2 cells leads to decreased iron and increased HIF-1α, which induces EMT in HK-2 cells.

## Discussion

**Figure 7:**
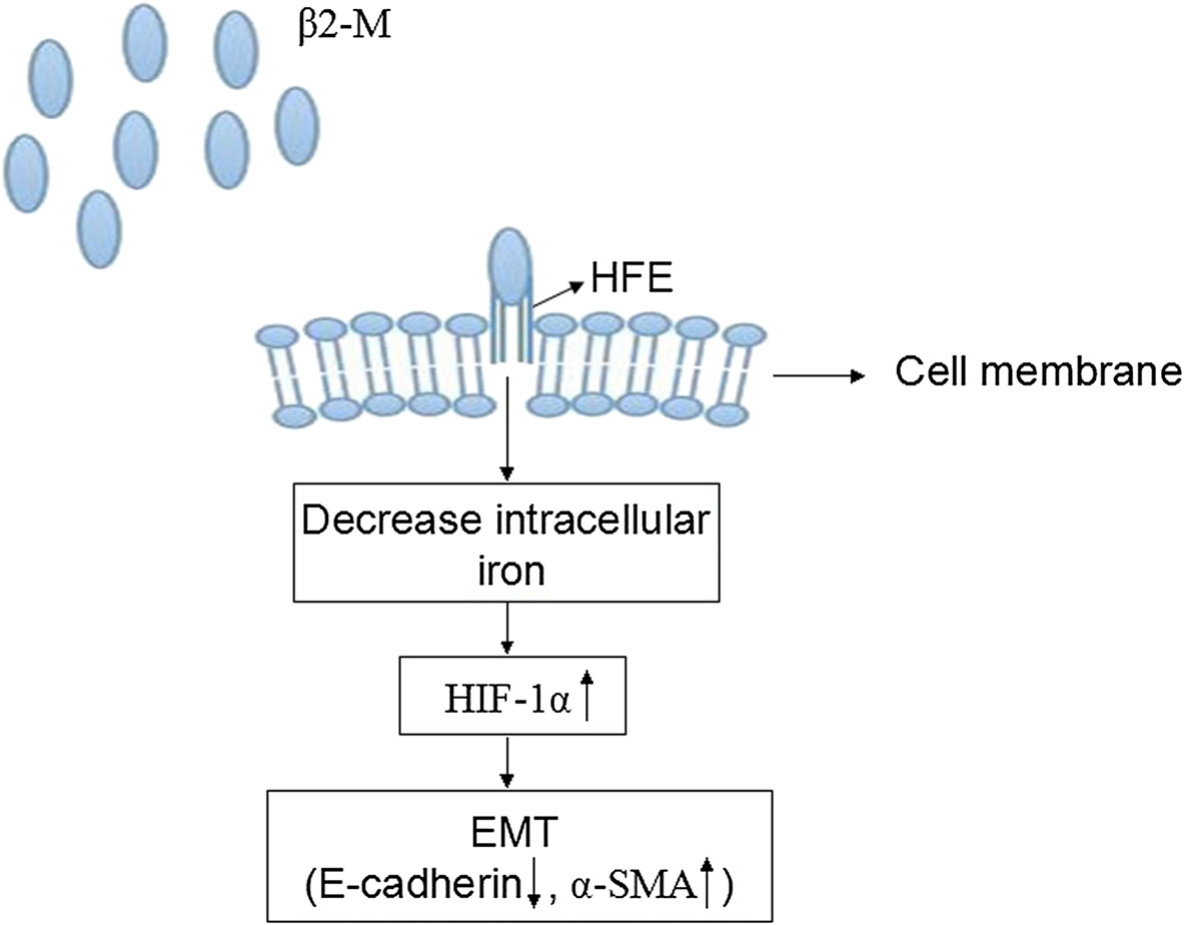
Working model of β2-M-mediated EMT. The β2-M/HFE complex maintains low iron levels and activates EMT via iron-responsive pathways, such as HIF-1α signaling.

The role of β2-M has long been documented in many kidney diseases. In this study, we documented for the first time that (i) β2-M can promote apoptosis and EMT in tubular epithelial cells *in vitro*; (ii) induced the stable expression of EMT biomarkers, including the decreased expression of E-cadherin and increased expression of α-SMA and fibronectin in HK-2 cells; and (iii) formed a complex with its receptor, HFE, and this complex regulated intracellular iron and activated HIF-1α in HK-2 cells (Figure 7). To our knowledge, this is the first report to demonstrate the manner by which β2-M functionally promotes EMT in tubular epithelial cells.

β2-M is a nonglycosylated protein with a molecular mass of 11,800 Da that is synthesized by all nucleated cells and forms a small invariable light chain subunit of major histocompatibility complex (MHC) class I antigen through noncovalent linkages on cell surfaces. The β2-M protein is present at low levels in serum, urine and other body fluids under normal physiological conditions and is almost exclusively catabolized within the kidney. Many studies have demonstrated that the serum or urine β2-M concentration is increased in a variety of diseases, including inflammatory or infectious diseases [10,11] and cancers [12-16]. When the elevation in plasma β2-M exceeds its renal reabsorptive threshold of 5 mg/1 or the proximal tubules are damaged, β2-M appears in the urine. In chronic kidney disease, as kidney function gradually declines, β2-M filtration is reduced and the serum level of β2-M is increased, which leads to the exposure of relatively normal residual glomeruli to high concentrations of β2-M. The excess β2-M filtered from the original urine can cause damage to renal tubular cells [18].

However, the actual concentration of β2-M in the proximal tubular ultrafiltrate in patients with proteinuric renal diseases is unknown. In systemic lupus erythematosus and chronic kidney disease patients, the maximum serum β2-M protein concentration has been reported to reach ~25 mg/dl [19,20]. Because the relatively small molecular weight of β2-M permits its filtration through the glomerular membrane, we chose to use the dose of 5–50 μM in our *in vitro* study. As shown in Figure 1, a significant decrease in cell viability (>20%) was observed at higher β2-M doses of ≥50 μM after 48 h. In addition, the induction of fibronectin, vimentin, and α-SMA expression by β2-M *in vitro* was dose dependent, and the maximal induction was observed following treatment with 25 μM of β2-M (data not shown). We therefore selected 25 μM of β2-M as the treatment in most of our cell experiments.

Although a conclusive demonstration of EMT *in vivo* in patients with kidney diseases remains a challenge, it has emerged as an important pathway leading to kidney fibrosis in diseased kidneys in recent years. The process of EMT is characterized by a loss of epithelial proteins, such as E-cadherin and cytokeratin, and the acquisition of new mesenchymal markers, including vimentin, α-SMA, fibroblast-specific protein-1 (FSP1), interstitial matrix components type I collagen, and fibronectin [21,22]. These alterations in protein expression are usually accompanied by a change in morphology to a fibroblastoid appearance and an enhanced migratory capacity. It has been proven that there is a correlation between the severity of tubular injury and urine protein quality in various renal diseases [23]. Additionally, Sajni Josson et al. [24] have found that β2-M, which is an important component of urine protein, induces epithelial to mesenchymal transition and confers cancer lethality and bone metastasis in human cancer cells. However, the molecular biological mechanism of β2-M in the damage of renal tubular epithelial cells is far from clear. In our study, β2-M induced tubular cells to undergo significant morphologic changes to a fibroblastoid appearance and promoted the expression of EMT-related proteins in these cells. These results indicate that this protein may also be an important factor in the peritubular microenvironment that induces or promotes EMT. Although we did not find any new molecular mechanism by which β2-M induces EMT, our results support the function of the β2-M/HFE complex as a mediator participating in β2-M-induced EMT in both renal proximal tubule epithelial cells and cancer cells. Further, this mechanism may play an important role in tumor-associated nephropathy because the concentrations of serum and urine β2-M have been shown to be increased in many types of cancer [13-17].

HFE is an MHC class 1b protein that is likely to assume a signaling role in association with β2-M [25]. β2-M/HFE has been found to regulate negatively intracellular iron, activate HIF-1α and promote EMT in cancer cells. Our study demonstrated that HFE is a β2-M receptor as shown by the following results: (1) HFE was found to physically interact with β2-M, as demonstrated by the immunoprecipitation results for the HK-2 cells (Figure 4A); and (2) the knockdown of HFE resulted in a reversal of EMT in the HK-2 cells with supportive morphologic, biochemical, and behavioral characteristics (Figure 4B-D). Thus, β2-M/HFE interactions are important for β2-M-mediated EMT. In addition, β2-M protected the influx and accumulation of intracellular iron. Higher β2-M/HFE levels led to the downregulation of intracellular iron in the HK-2 cells, and low levels of this complex in these cells enhanced intracellular iron levels (Figure 5A). Lower levels of intracellular iron activated HIF-1α and its target genes in the HK-2 cells (Figures 5B-C and 6), driving EMT. The downstream functional significance of the β2-M/HFE complex is depicted in Figure 7.

## Conclusion

In summary, we demonstrated the importance of β2-M in EMT in tubular epithelial cells. The activity of β2-M is mediated by the β2-M/HFE complex, which regulates intracellular iron homeostasis and HIF-1α and ultimately induces EMT. The inhibition of this complex could affect the process of EMT.

## Abbreviations

β2-M: β2-microglobulin
EMT: Epithelial-mesenchymal transition
PTEC: Proximal tubular epithelial cells
α-SMA: α-smooth muscle actin
HFE: Hemochromatosis
HIF-1α: Hypoxia inducible factor-1α
